# *TNC* and *GJA1* Are Putative Progenitor Markers That Are Localized in the Perivascular Adventitia of the Adult Monkey Brain Subventricular Niche

**DOI:** 10.3390/ijms26041397

**Published:** 2025-02-07

**Authors:** Martin N. Ivanov, Dimo S. Stoyanov, Lora V. Veleva, Andon M. Mladenov, Stoyan P. Pavlov, Tetsumori Yamashima, Anton B. Tonchev

**Affiliations:** 1 Department of Anatomy and Cell Biology, Faculty of Medicine, Medical University, 9000 Varna, Bulgaria; dimo.stoyanov@mu-varna.bg (D.S.S.); lora.veleva@mu-varna.bg (L.V.V.); andonmladenov5@gmail.com (A.M.M.); stoyan.pavlov@mu-varna.bg (S.P.P.); 2 Department of Stem Cell Biology, Research Institute, Medical University, 9000 Varna, Bulgaria; 3 Department of Neurosurgery, Division of Neuroscience, Kanazawa University Graduate School of Medical Science, Kanazawa 920-8641, Japan; yamashima215@gmail.com

**Keywords:** *TNC*, *GJA1*, SVZ, neurogenesis, markers

## Abstract

The largest area in the adult mammalian brain that contains stem and progenitor cells at different stages of differentiation is the subventricular zone located along the lateral wall of the lateral ventricle. We have previously shown in adult monkeys that transient global cerebral ischemia upregulates the expression of hundreds of genes in this zone, including genes known to be related to stemness in the rodent brain. Here, we analyzed the immunophenotype of two of these genes, *TNC* and *GJA1*, by co-expression experiments, applying a panel of known stem/progenitor-cell-related markers. We found that both *TNC* and *GJA1* were expressed in the perivascular region. They were localized not to the endothelial cells but to the periendothelial adventitial cells, which was consistent with our previous electron-microscopic data suggesting periendothelial cells as a source of progenitors. We report that the expression of *GJA1* was high in quiescent progenitors, while *TNC* was mostly present in progenitors in the transition from a quiescent to an active state. Our data suggest that *TNC* and *GJA1* can be used as markers for stem/progenitor cells in the largest stem cell area of the adult primate brain.

## 1. Introduction

The process by which new neurons are produced by stem/progenitor cells in the adult brain is known as adult neurogenesis. This process occurs in specialized histological domains known as neurogenic niches: the subventricular zone of the lateral ventricle (SVZ) and the subgranular zone (SGZ) of the dentate gyrus [1,2,3]. The presence of proliferating and differentiating cells in these niches is widely studied in rodents and less studied in primates. The neural stem cells (NSCs) in the brain of mammals exist in different stages of development, such as active NSCs (aNSCs) or quiescent NSCs (qNSCs) [4,5]. Rarely dividing and inactive under physiological conditions, qNSCs can transform into aNSCs after stimulation, and as aNSCs, become highly proliferative and exhibit distinct differences in their immunophenotypic characteristics [6]. In particular, aNSCs begin to express the Epidermal Growth Factor Receptor (EGFR). qNSCs do not express proliferation indicator proteins such as Ki67 (a marker expressed during the G1 phase of the cell cycle) [7]. The investigation of the presence and proliferation of neural stem cells and their progeny in the adult primate, including humans, brain is slowed down by the lack of reliable markers for these cells [8,9,10,11,12]. Thus, environmental triggers that can stimulate qNSC to adopt an activated state are useful when studying the qNSC-to-aNSC transition. Ischemic stroke is caused by the thromboembolic blockage of a major cerebral blood vessel or one of its branches. The occlusion of a cerebral vessel leads to blood and oxygen deprivation. This condition is an established trigger of increased brain progenitor cell proliferation in primates [13,14]. A public digital database (www.monkey-niche.org) shows the expression changes in 150 genes that accompany the post-ischemic increase in NSC proliferation in the adult primate SVZ [15]. Here, we visually inspected the images of the 150 genes included in the database and selected 2 candidate genes: *TNC* and *GJA1*. We provide evidence suggesting that *TNC* and *GJA1* are candidate genes that mark stem and progenitor cells in the normal adult monkey anterior SVZ (SVZa).

TNC (Tenascin-C) is an extracellular protein found around neurons and glia in the CNS and also in stem cell niches in the intestinal crypts, bone marrow, and hair follicles. Its presence has been described in various pathological conditions, namely, tumor masses, inflammation, and mechanical and chemical injuries, with its expression being enhanced during tissue regeneration [16,17]. As part of the extracellular matrix (ECM), TNC is directly involved in adhesion to other ECM proteins like fibronectin, integrin, collagen, periostin, fibrillin-2, and others [16,17]. TNC has the ability to induce EGFR expression, thus stimulating cell proliferation [18]. TNC interacts with factors like TGFβ (Transforming Growth Factor beta), Wnt3a (Wingless 3a), and VEGF (Vascular Endothelial Growth Factor). TNC is known to be expressed in the rodent neurogenic niche, limited to the subependymal layer (SEL) of the SVZ and rostral migratory stream (RMS) [17]. In the rodent SVZ, TNC expression was detected in GFAP+ cells, most likely qNSCs, but not in cells expressing markers like PSA-NCAM (Polysialylated -–Neural Cell Adhesion Molecule), marking neuroblasts, and Ascl1 (Achaete-scute homolog 1), marking transit-amplifying progenitor cells (TAPs) [19].

GJA1 (Connexin 43) is a transmembrane protein that plays a crucial role in intercellular communication. It is expressed in various tissues and organs, including the brain. GJA1 is expressed in both embryonic and adult subventricular zones, where it plays a role in maintaining the proliferative abilities of NSCs. The elevated expression of GJA1 is sufficient to stimulate the formation of functional extracellular channels, which is an important condition for maintaining and proliferating NSCs. Additionally, the levels of GJA1 decrease as NSCs begin to differentiate into neurons [20,21].

Our previous transcriptomic analysis revealed the significant upregulation of *TNC* and *GJA1* in the SVZ following transient global cerebral ischemia. This selective upregulation points to their relevance in the primate SVZ microenvironment and their potential as key markers for progenitor cell identification [15]. Furthermore, we utilized our bioinformatic analysis of the transcriptome data and compared it to available SVZ transcriptomes. When making this comparison with Dulken et al., we found that *GJA1* is enriched in the categories of astrocytes and qNSC-like cells, while TNC was enriched within the astrocyte category [7]. When comparing our data with Llorens-Bobadilla et al.’s dataset, we found that *GJA1* was enriched in primed qNSCs [5]. Colorimetric in situ hybridization (ISH) staining showed that these two genes were markedly induced in the subependymal region of the SVZa that hosts the stem progenitor cells in the primate brain [15]. In particular, *TNC* and *GJA1* expression was common in perivascular adventitial cells that represent putative progenitors in the primate SGZ neurogenic niche [22]. In the present study, we performed fluorescent in situ hybridization (FISH) for each of the selected genes followed by the immunohistochemical co-labeling of markers for proliferation and known cell types in order to probe the putative expression of *TNC* and *GJA1* in stem/progenitor cells in intact monkey brains. We studied the expression of these genes before and after cerebral ischemia, which is known to induce SVZ progenitor cell proliferation, and combined these expression studies with immunolabeling for known markers of brain cell types. Our selection of *TNC* and *GJA1* as candidate markers for neural stem/progenitor cells in the primate SVZ is supported by their distinct functional roles, spatial localization, and relevance to neurogenic processes. By highlighting these markers’ specific contributions to identifying and characterizing progenitor populations, we aim to advance the understanding of stem cell biology in the adult primate brain and bridge interspecies differences in neurogenesis research. Our results demonstrate that a combinatorial labeling strategy may prove an effective method in the visualization of neural stem/progenitor cells in the adult primate brain.

## 2. Results

### 2.1. TNC and GJA1 Expression in the SVZa Before or After Cerebral Ischemia

**Figure 1:**
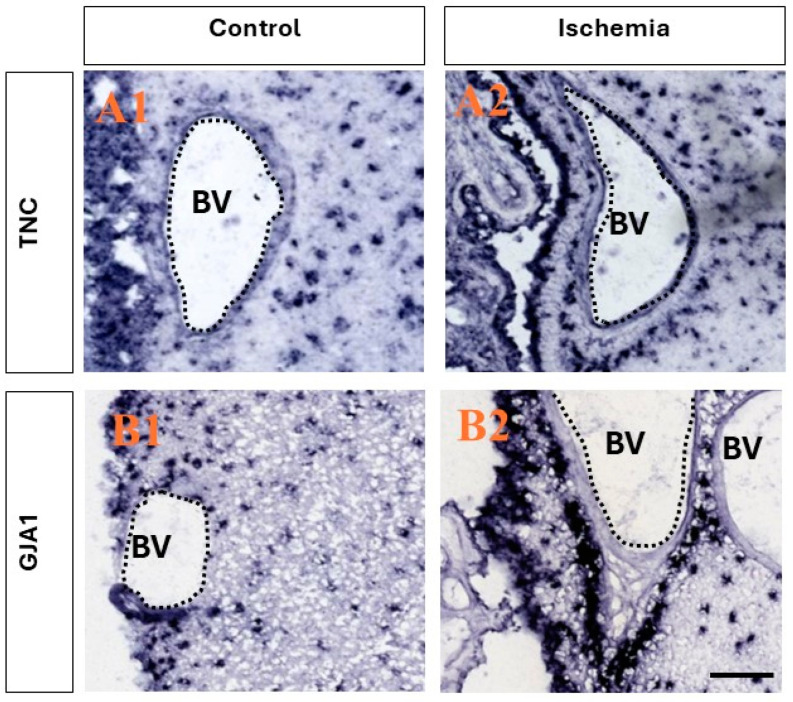
Ischemia-induced upregulation of the expression of *TNC* and *GJA1* in adult monkey neurogenic niche. (**A1**,**A2**) Comparison between the expression of *TNC* before and after ischemia. After ischemia, a stronger presence is seen in both the ependymal layers and in the SVZ, and the clustering of positive *TNC+* cells is observed in close association with the blood vessels in the region, while little to no expression is seen in the epithelial cells lining the blood vessels. (**B1**,**B2**) Comparison between the expression of *GJA1* before and after ischemia. After ischemic injury, an increase in expression in the ependymal layers and SVZ is observed, and intense staining is observed in close association with the blood vessels, with no staining in the epithelial cells lining the blood vessels. Images derived from www.monkey-niche.org. Scale bar—200 μm. BV—blood vessel.

Utilizing a publicly available database (www.monkey-niche.org accessed on 18 February 2024) of gene expression before or after brain ischemia in the adult monkey SVZ, we noticed that *TNC* and *GJA1* were markedly increased after ischemia in the subependymal region that hosts the stem/progenitor cells in the primate brain [15]. Moreover, the selected two genes also demonstrated a marked increase in expression with a distinct clustering of the cells in the SVZ and, more notably, perivascularly (Figure 1), which is characteristic of progenitors [22]. It should be noted that the endothelial cells on the SVZ blood vessels (BVs) did not seem to be positive for either *TNC* or *GJA1*.

### 2.2. Immunophenotype of TNC-Expressing Cells

Immunohistochemical staining for GFAP, VIM, and BrdU was conducted in combination with FISH for *TNC*. First, we calculated the fraction of double-positive (*TNC*+/marker+) cells of all SVZ cells (marked by DAPI). Our staining showed that *TNC*+/GFAP+ cells and *TNC*+/VIM+ cells represented approximately a quarter of the cells in the SVZa, while *TNC*+/BrdU+ were only 1.4% of the cells in the SVZa.

**Figure 2:**
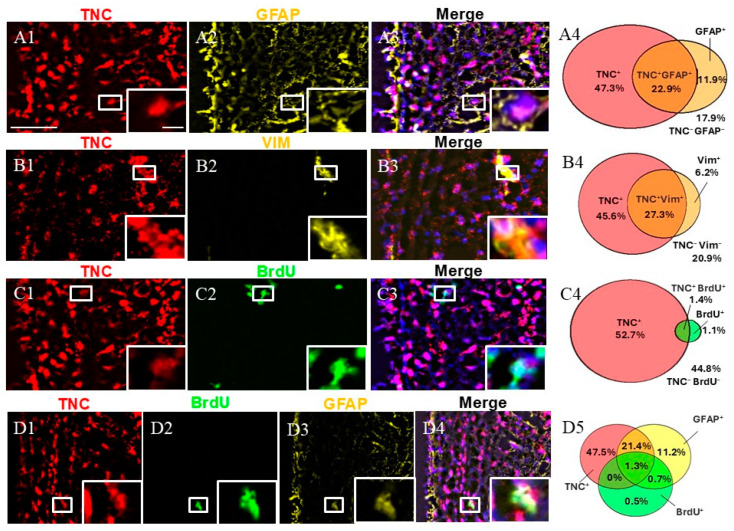
Expression pattern of *TNC+* cells in adult monkey SVZa. (**A1**–**A3**) Dual staining for *TNC* (red) and GFAP (yellow) counterstained with DAPI. *TNC*/GFAP co-staining demonstrates the presence of *TNC*+/GFAP+ cells (insert). (**B1**–**B3**) Dual staining for *TNC* (red) and VIM (yellow). *TNC*/VIM co-staining reveals the presence of *TNC*+/VIM+ cells (insert). (**C1**–**C3**) Dual staining for *TNC* (red) and BrdU (green). *TNC*/VIM co-staining shows the presence of *TNC*+/BrdU+ cells (insert). (**D1**–**D4**) Triple staining for *TNC* (red), GFAP (yellow), and BrdU (green) demonstrates the presence of the proliferative NSCs (*TNC*+/GFAP+/BrdU+; insert). (**A4**,**B4**,**C4**,**D5**) Venn diagrams depicting the share of different cell subpopulations. The percentages reflect the fraction of a specific cellular expression pattern of all cells in the SVZ as stained by DAPI. Scale bar—100 μm. Scale bar insert—20 μm.

We studied the fraction of GFAP+ cells expressing *TNC*. GFAP is a marker for qNSC/ parenchymal astrocytes [4,7]. We discovered that while two-thirds of the GFAP+ cells co-expressed *TNC* (95% CI 60.45–74.42%), only one-third of the *TNC*+ cells were co-labeled for GFAP (95% CI 28.15–37.87%) (a total of 1158 counted cells) (Figure 2). We also studied the fraction of VIM+ cells that were co-labeled for *TNC*. VIM is a marker for aNSCs, TAPs, and blood vessels [15]. We found that 27% of the *TNC*+ cells were positive for VIM (95% CI 29.96–43.52%), while 81% of the VIM+ cells expressed *TNC* (95% CI 74.45–90.42%) (a total of 716 counted cells). To track and quantify de novo generated cells in this area, we injected monkeys with BrdU [13,14] followed by immunostaining. Double-labeling *TNC*/BrdU experiments showed that 3% of all *TNC*+ cells were BrdU-positive (95% CI 1.67–3.61%), while 56% of all BrdU+ cells were *TNC*-positive (95% CI 43.52–71.93%) (a total of 4244 counted cells). To differentiate between qNSCs and aNSCs, we used triple staining, including GFAP and BrdU in combination with *TNC*. This revealed the existence of a subpopulation of *TNC+/*GFAP+/BrdU+, 1.3% of all DAPI+ cells, representing NSCs initiating proliferation (Figure 2(D4)). In contrast, the non-proliferative (quiescent) qNSC fraction (*TNC*+/GFAP+/BrdU−) was estimated to be 21.4% of all SVZa cells (Figure 2(D4)).

### 2.3. Immunophenotype of GJA1-Expressing Cells

**Figure 3:**
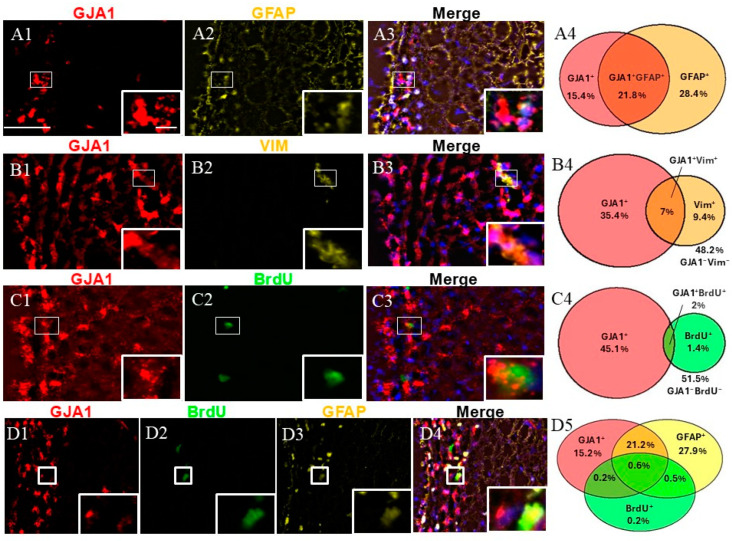
Cell identity of *GJA1+* cells in adult monkey rostral SVZ. (**A1**–**A3**) Dual staining for *GJA1* (red) and GFAP (yellow). *GJA1*/GFAP co-staining demonstrates the presence of *GJA1*+/GFAP+ cells (insert). (**B1**–**B3**) Dual staining for *GJA1* (red) and VIM (yellow) reveals the presence of *GJA1*+/VIM+ cells (insert). (**C1**–**C3**) Dual staining for *GJA1* (red) and BrdU (green). *GJA1*/BrdU co-staining reveals the presence of proliferative cells *GJA1*+/BrdU+ (insert). (**D1**–**D4**) Triple staining for *GJA1* (red), GFAP (yellow), and BrdU (green). *GJA1*/GFAP/BrdU co-staining demonstrates the presence of proliferative NSCs (*GJA1*+GFAP+BrdU+, insert). (**A4**,**B4**,**C4**,**D5**) Venn diagrams depicting the different cell subpopulations. The percentages reflect the fraction of a specific cellular expression pattern of all cells in the SVZ as stained by DAPI. Scale bar—100 μm. Scale bar insert—20 μm.

An immunophenotypic analysis of *GJA1*+ cells was performed using the markers GFAP, VIM, and BrdU (Figure 3). Dual labeling revealed that while *GJA1*+/GFAP+ represented approximately a quarter of the SVZ cells (Figure 3(A4)), *GJA1*+/VIM+ and *GJA1*+/BrdU+ were less than 10% of the SVZ cells (Figure 3(B4,C4)).

We studied the fraction of GFAP+ cells expressing *GJA1*. More than half of the *GJA1*+ fraction co-expressed GFAP (95% confidence interval: 45.92–60.64%), and again, nearly half of the GFAP+ cells co-expressed *GJA1* (95% confidence interval: 34.26–47.04%) (a total of 1033 counted cells). The evaluation of *GJA1*/VIM showed that 16.4% of all *GJA1*+ cells co-expressed VIM (95% confidence interval: 14.01–20.60%), and nearly half (42%) of the VIM+ cells co-expressed *GJA1* (95% confidence interval: 33.40–53.78%) (a total of 1250 counted cells). Additionally, 4% of all BrdU+ cells co-expressed *GJA1* (95% confidence interval: 2.46–5.72), whereas more than half (57.6%) of all *GJA1*+ cells co-expressed BrdU (95% confidence interval: 43.33–73.52%) (a total of 2717 counted cells). Finally, when we evaluated a triple combination of BrdU, GFAP, and *GJA1*, our results revealed that 0.6% of all cells were triple-positive (*GJA1*+/GFAP+/BrdU+), representing NSCs in a proliferative state, while 21.1% of the cells were *GJA1*+/GFAP+/BrdU−, thus representing a quiescent state (a total of 1033 counted cells).

### 2.4. Dual Labeling for TNC and GJA1

**Figure 4:**
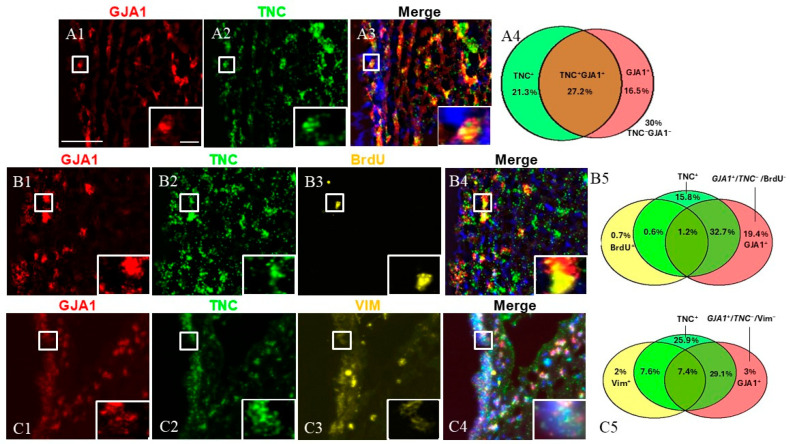
SVZa cell subpopulations expressing *TNC* and *GJA1*. (**A1**–**A3**) Dual FISH staining for *TNC* (green) and *GJA1* (red). A *TNC*+/*GJA1*+ cell is shown in the insert. (**B1**–**B4**) Dual FISH staining for *TNC* (green) and *GJA1* (red) in combination with BrdU (yellow) demonstrates the presence of a *GJA1*+/*TNC*−/BrdU− population (**C1**–**C4**). Dual FISH staining for *TNC* (green) and *GJA1* (red) in combination with VIM (yellow) demonstrates the presence of a *GJA1*+/*TNC*−/VIM−population (insert). (**A4**,**B5**,**C5**) Venn diagrams depicting the different cell subpopulations. The percentages reflect the fraction of a specific cellular pattern of all SVZa cells (stained by DAPI). Scale bar: 100 μm. Scale bar insert: 20 μm.

Next, we investigated the cells expressing both *TNC* and *GJA1* (Figure 4). To this end, we utilized dual FISH staining for *TNC* and *GJA1* and found that 27.2% of all studied SVZ cells were *TNC*+/*GJA1*+, while 21.3% were *TNC*+/*GJA1*− and 16.5% were *TNC*−/*GJA1*+ (Figure 4(A1–A4)) (a total of 2540 counted cells). To investigate deeper the expression pattern of these cell subpopulations, we combined *TNC*/*GJA1* dual FISH with immunohistochemical staining for BrdU or VIM (Figure 4(B1–C5)). We discovered that triple-positive *TNC*+/*GJA1*+/BrdU+ cells represented a very small (~1%) fraction of the SVZ cells (Figure 4(B5)) (a total of 1041 counted cells), while the *TNC*+/*GJA1*+/VIM+ cells accounted for 7–8% of the SVZ cells (Figure 4(C5)) (a total of 716 counted cells). Interestingly, we noticed the presence of a significant subpopulation of SVZ cells that were *GJA1*+/*TNC*−(~25% of the SVZ cells), and these cells expressed neither BrdU (Figure 4(B5)) nor VIM (Figure 4(C5)).

## 3. Discussion

The level of neurogenesis varies among species, with it being lower in primates compared to the commonly used rodent models [23]. Furthermore, research on adult neurogenesis in non-human primates offers limitations due to technological and ethical constraints [24,25,26]. Of note, the structural organization of the NSC niche shows interspecies differences between rodents and primates [27]. Thus, the identification of markers of progenitor cell populations in the stem cell niche of primates is of pivotal importance in tracking these cells and being able to identify them in their complex environment in the niche [28,29].

Aging is a critical factor influencing neurogenesis and the expression of key markers in the primate SVZ. Studies have shown that *TNC* expression decreases with age in tissues such as skin, cartilage, and the cardiovascular system. This downregulation is linked to reduced regenerative capacity and extracellular matrix remodeling in conditions like fibrosis and cardiovascular diseases [30,31]. This response may exacerbate maladaptive remodeling and inflammation. Aging is also associated with decreased *GJA1* expression in many tissues, impairing gap-junction-mediated communication [32]. Aging impacts the phosphorylation, localization, and turnover of *GJA1*, reducing its effectiveness [33]. Age-related oxidative stress and inflammation exacerbate *GJA1* dysfunction, particularly in the nervous system [34].

In addition to the expression of *TNC* and *GJA1*, various signaling pathways, such as BMP, WNT, and NOTCH, are critical in regulating neurogenesis and progenitor cell behavior in the adult brain, particularly in response to injury or aging [35,36]. NOTCH signaling, known for maintaining progenitor cell quiescence, likely plays a role in regulating the activation of cells expressing *TNC* and *GJA1* [37]. BMP signaling, which is involved in cell differentiation and repair, might modulate the transition of progenitors from quiescence to an active state [38]. Meanwhile, WNT signaling is crucial for progenitor cell proliferation and migration, which could be particularly relevant in ischemic injury models where tissue regeneration is required [39]. The interactions between these pathways, particularly in the context of *TNC* and *GJA1* expression, might dictate the behavior of progenitor cells and their response to injury, including ischemia.

Further studies exploring how these pathways interact with *TNC* and *GJA1* in the primate SVZ will be important to understand how cellular behaviors such as activation, proliferation, and differentiation are regulated in the stem cell niche of primates.

Here, we report for the first time the cellular immunophenotypic characteristics of cells expressing *TNC* and *GJA1* in the adult primate SVZ. Our findings show that in the normal adult monkey SVZ, *TNC* and *GJA1* are expressed by both qNSCs and aNSCs. Our combined FISH/immunofluorescent analyses revealed two subpopulations of *TNC*+ cells: GFAP+/*TNC*+/BrdU− (putative qNSCs) and GFAP+/*TNC*+/BrdU+ (putative aNSCs). Thus, while *TNC* is expressed by stem cells in both rodents and monkeys, in monkeys, *TNC* is also expressed by the aNSC/TAP (*TNC*+/VIM+) population of progenitors. In the rodent SVZ, *TNC* expression is restricted to GFAP+ cells [19], and thus, our data showing that *TNC* expression is maintained in a primate aNSC/TAP population suggest an interspecies difference.

Similarly to *TNC*, we detected two subpopulations of *GJA1*+ cells: GFAP+/*GJA1*+/BrdU− (putative qNSCs) and GFAP+/*GJA1*+/BrdU+ (putative aNSCs). GJA1 has a role in maintaining the proliferation and self-renewal of NSCs in mice. This is in line with our data, which may represent an interspecies similarity in the stem cell biology of the SVZ. We also observed a distinct decrease in gene expression when cells gradually differentiated from qNSCs to aNSCs in the two examined genes.

Additionally, using co-staining with *TNC* and *GJA1*, we discovered a subpopulation of *GJA1*+/*TNC*− cells, which was negative for both VIM and BrdU (Figure 4). The *GJA1*+/*TNC*− expression pattern most likely corresponds to a quiescent progenitor or to niche astrocytes.

A present limitation in brain stem cell research is the fact that the currently used markers can label more than one type of cellular subpopulation. For example, GFAP can label both qNSCs and niche astrocytes. Similarly, VIM and BrdU can label more than one cell population. In the current study, we chose a different starting point when focusing on the genes of interest: we used cerebral ischemia as a tool to trigger stem cell activation, and, at the same time, we searched for genes with a specific perivascular expression pattern, as such a pattern was proved to label progenitors in the hippocampal niche [22]. Furthermore, we employed a triple-labeling combining FISH with dual immunofluorescence approach to combine the gene of interest with the expression of not one but two different markers, in combinations. This combinatorial labeling allowed us to distinguish between qNSCs (GFAP+/VIM−/BrdU−) or aNSCs (GFAP−/VIM+/BrdU+) and define the fractions of these progenitors that express *TNC* and *GJA1*. An important step in differentiating qNSCs from niche astrocytes emerged from our effort to better characterize the molecular features of *TNC*− and *GJA1*− expressing cells. One putative fraction representing the niche astrocytes is the population of *GJA1*+/*TNC*− cells that we observed. Future research may clarify the molecular mechanisms by which cerebral ischemia regulates the expression of *TNC* and *GJA1*.

## 4. Materials and Methods

For our experiments, we used tissues from 4 adult (four–six years old at the time of the experiment) macaque monkeys (Macaca fuscata). The tissues were derived following an experimental procedure approved by the Animal Care and Ethics Committee of Kanazawa University, Japan (approval protocols #AP-031498 and #AP-080920). Ischemia was induced using surgical procedures described previously [13,14]. To induce global ischemia, each monkey was anesthetized (ketamine at a dose of 2–5 mg/kg, i.m.), intubated, and connected to a ventilator. During the surgical procedures, the monkeys were additionally anesthetized via inhalation (1% halothane, gas mixture 40% O_2_/60% N_2_O). Arterial blood pressure, pulse, pupil diameter, and response were monitored. During the operation, the animals’ body temperature was maintained within 37 ± 0.5 °C. The surgical procedure for global ischemia was performed under sterile conditions in the following sequence: anterior median thoracotomy, the dissection of the skin and subcutaneous tissue, sternotomy, the dissection of soft tissues, and the visualization of the left subclavian artery and brachiocephalic trunk. One of the four monkeys received 5-bromo-2’-deoxyuridine (BrdU 100 mg/kg, i.v., from Sigma-Aldrich Corp., St. Louis, MO, USA) for 5 days and was sacrificed 2 h after the last BrdU application. Coronal sections of the anterior subventricular zone (SVZa) were cut on a freezing microtome (Leica CM3050 S, Leica Biosystems, Deer Park, IL, USA) at 20 micrometers. The non-radioactive in situ hybridization (ISH) staining procedure and the combinatorial labeling protocol with cell type markers have been published previously [15]. Briefly, macaque monkey-specific templates, 600–900 nucleotides long, were synthesized using RNA isolation, cDNA synthesis, and PCR amplification. The templates were generated using the following specific primers—*TNC* Forward: CAGAG-GAAGGAGCTCGCTA; *TNC* Reverse: GACACCAGGTTCTCCAGCTC; *GJA1* Forward: AGCCTACTCAACTGCTGGAG; and *GJA1* Reverse: TCGCCAGTAACCAGCTTGTA. The primer and template sequences used for the ISH of the genes *TNC* and *GJA1* were identical to those published at www.monkey-niche.org [20]. In vitro transcription was used to generate Digoxigenin-tagged riboprobes. Hybridized probes were identified using a two-step chromogenic catalyzed reporter deposition method. For FISH staining, the *TNC* and *GJA1* hybridized probe was detected with fluorochrome-labeled reagents. Following fluorescent ISH (FISH), sections were subjected to antigen retrieval (Dako PT Link, Agilent Technologies, Santa Clara, CA, USA) with a citrate buffer (pH 6) and blocked with Bovine serum albumin diluted 1:10 in 1% of Triton X-100/PBS. We used the following primary antibodies: anti-BrdU (1:100, Cat. No Ab6326, Abcam, Cambridge, UK), anti-GFAP (1:400 Cat. No M0761, Dako-Agilent Technologies GmbH, Hamburg, Germany), anti-GFAP (1:1000, Cat. No AB5541, Merck Millipore, Burlington, MA, USA), and anti-VIM (1:1000, Cat. No MAB3400, Merck Millipore, Burlington, MA, USA). The primary antibodies were visualized by species-specific secondary antibodies, counterstained with DAPI and covered with mountant media (Invitrogen™ ProLong™ Gold Antifade Mountant, Thermo Fisher Scientific, Waltham, MA, USA). A high-resolution mosaic image of the periventricular tissues was captured using an EC Plan-Neofluar 20×/0.50 objective with a lateral resolution of 0.65 µm/px. These images serve as a reference database and are intended for future analyses. A series of 3 to 5 z-stacks per microscope slide were obtained from the SVZ using an EC Plan-Neofluar 40×/0.75 objective, with a lateral resolution of 0.325 µm/px and an axial resolution (z-distance) ranging from 0.125 to 0.55 µm. The camera was set to a binning factor of 2 × 2 to reduce both scanning time and camera noise. The z-stacks from the striatal SVZ were captured sequentially, starting from the dorsolateral edge of the ventricle and progressing medially. The co-localization quantification of cells labeled by fluorescent immunohistochemistry was conducted as described previously [15]. Briefly, using a semi-automated digital workflow, we processed the acquired images by channel extraction and denoising followed by the top-hat filtering, channel thresholding, classification, and counting of the cell in a region of interest (1000 cells per specimen).

## Data Availability

All data are contained within the article.
